# GIANT PITYRIASIS ROSEA

**DOI:** 10.4103/0019-5154.62750

**Published:** 2010

**Authors:** Vijay Zawar

**Affiliations:** *From the Shreeram Sankul, Opp. Hotel Panchavati, Vakilwadi, Nashik-422 001, Maharashtra, India.*

**Keywords:** *Atypical pityriasis rosea*, *dermatophytosis*, *gigantic pityriasis rosea*

## Abstract

Pityriasis rosea is a frequent papulo-squamous disease and is known for various atypical clinical presentations. We report an adult female patient with a clinical diagnosis of giant pityriasis rosea, which is a rarity in clinical practice.

## Introduction

Pityriasis rosea (PR) is a common papulo-squamous disorder characterized by an onset as a herald plaque and followed by a multiple oval to round smaller scaly secondary eruptions delineating to lines of cleavage. Current evidence indicates that PR is a type of viral exanthema and the etiology may be possibly linked to human herpes viruses.[1–3]

While most cases present the typical pattern clinically, there are about 20% patients of PR presenting in a deviated clinical appearance and might pose a diagnostic problem[2–5]

Several unusual variants are reported in the literature[3–6] including unilateral,[5] inverse,[3] lichenoid,[4] vesicular, papular,[7,8] purpuric,[7,9] hemorrhagic,[10] erythema multiforme-like,[11] urticarial[7] and those involving mucosae,[12] palms and soles, flexures, face and, may even be generalized having exfoliative dermatitis.[7]

## Case Report

A 35-year-old housewife presented with mildly pruritic scaly eruptions on the back and front of chest for 2 weeks. Earlier, she had a single large similar eruption on left breast 10 days ago. She gave a history of upper respiratory infection 2 weeks before the onset of first lesion, when she had a mild fever, coryza, and malaise lasting for 5 days. She received a combination of ibuprofen+paracetamol and cetrizine orally, prescribed by a family practitioner for 3 days. She had been treated with the same drugs several times earlier by the same physician.

There was no history of similar lesions in the past. Her past and family health was unremarkable.

There was no history suggestive of allergic or irritant contact dermatitis in the present case. She did not receive any other systemic medications in the recent past. Travel history was insignificant. She distinctly denied a history of tick bites.

The first lesion was a large oval plaque measuring approximately 7 cm × 6 cm, almost occupying the whole left breast, with peripheral collarette scaling and central clearing with minimal itching. She was treated by a family physician with topical miconazole cream for 10 days without significant resolution. The patient refused the front of chest to be photographed and hence, a picture of herald plaque could not be taken. Prescriptions brought by the patient were verified specifically and did not contain topical or systemic steroids.

**Figure 1:**
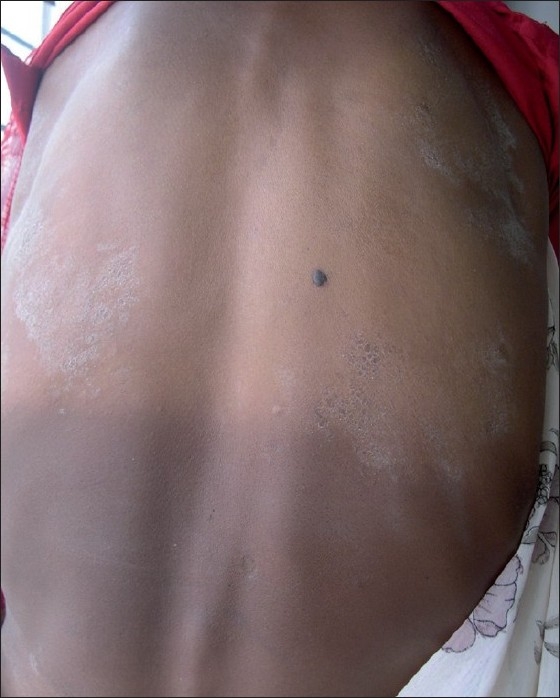
Multiple, sharply demarcated, large scaly lesions of irregular shape on the back of trunk with peripheral scaling and central clearing. One plaque on right upper back looked parallel to the cleavage lines. Collarette scaling was visible at few places. One lesion on left upper back was pear-shaped

**Figure 2:**
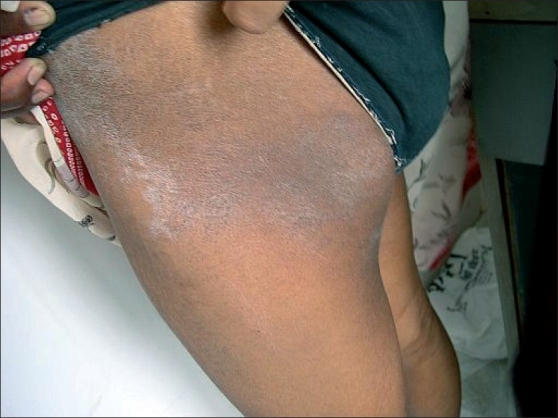
Sharply demarcated, large scaly plaque on the lateral thigh extending upto the hip. A similar plaque was also present on contralateral side in a symmetrical distribution

She suddenly developed the subsequent lesions as multiple, sharply demarcated, large scaly lesions of irregular shape on front and back of trunk as well as lateral thighs extending upto the hips on both sides somewhat in symmetrical distribution. The size of individual lesions varied from 5 to 7cm in longest diameters. The periphery of few of these lesions still showed collarette scaling at places. Only the plaque on right upper back showed to be placed along the line of cleavage and others were not so classical [Figure 1, 2].

All the eruptions consisted of scaly plaques with central clearing and peripheral scales. Collarette scaling was seen at places on the affected areas. There palms and soles and mucosal surfaces were uninvolved.

Her general and systemic examinations revealed no abnormality. Investigations including complete blood counts, blood sugar levels, urinalysis, HIV antibodies, and VDRL test (done in repeated dilutions) were negative or normal. The fungal scrapings were repeated twice and did not reveal any evidence of fungal elements. She did not, however, agree for skin biopsy.

She was prescribed topical betamethasone dipropionate 0.025% ointment twice a day topical application and desloratidine tablet 5 mg once a day. The lesions slowly resolved within 2 weeks, with slight hypopigmentation. There was no recurrence for 6 months of follow up.

## Discussion

Atypical or unusual presentations in PR are often seen in clinical practice. Diagnostic dilemma persists in such patients unless a careful clinical observation and follow up are made.[1–3,6] Such patients may often be over-investigated. Moreover, being the disease not much bothersome with a tendency of self-resolution, the patients are often lost to follow up and remain undiagnosed.

Considering clinical course, typical herald plaque with collarette scale at the initial lesion and complete resolution within 3 weeks, we believe this case deserves a diagnostic label of gigantic PR. Other possibilities such as secondary syphilis, pityriasis lichenoides, erythema annulare centrifugum, erythema chronicum migrans, tinea corporis, psoriasis, and drug-induced PR were unlikely in our patient. Multiple herald plaques may cause diagnostic confusion in such situations. However, even this was not likely in our case considering primary lesion on left breast 10 days earlier to sudden onset of secondary lesions. Moreover, classical collarette scaling on the breast and at places on the truncal and thigh lesions and resolution within a span of few weeks without recurrence further supports our view of gigantic PR.

Scaly annular, larger eruptions are known to occur in another rare variant known as PR of Vidal, which presents at limb girdles involving axilla and inguinocrural areas.[1–3,6] Our patient did not have any lesions in these areas. PR-like eruptions are reported after ingestion of anti-inflammatory and antipyretic drugs.[13] But, a detailed history and follow up in our patient did not point to other alternative diagnoses such as drug eruption, dermatophytosis, and contact dermatitis. Unfortunately, skin biopsy was not possible in our case. However, histopathological features are generally not diagnostic in PR and they usually presents with non-specific dermatitis. Skin biopsy is not routinely performed for diagnosis of PR in India. It may be helpful to rule out other diagnosis when in doubt. In our experience, in the absence of skin biopsy, a meticulous follow up of clinical course in the given patient is helpful in arriving at a proper diagnosis.

Gigantic PR is rarely reported in the literature and was named after Darier. In his proposed clinical classification in 1924, Klauder described PR gigantean into confluent and diffuse variant according to morphology.[14] Pringle earlier described that PR gigantic is very rare and consists of plaques and circles of very large size wherein the individual lesions may reach the size of palm of the patient.[14] The size he described ranged between 5 cm and 6.3 cm. One of the lesions on the left side of back in our patient appeared as pear shaped, as described in Pringle's report. The clinical course in our patient was similar to the classical PR, as suggested by Pringle.[15]

Our case illustrates that PR can present as multiple, large scaly plaques. In such patients, good clinical observation and follow up are keys to diagnosis. Without knowledge of this entity, the diagnosis may be missed. Clinicians need to be alerted to this rare variant of PR. This case is being reported here for its extreme rarity. To the best of our knowledge, this is the first report of gigantic PR in the Indian literature. The variant may be under-reported.
